# Recurrent Intracerebral Hemorrhage in a 30‐Year‐Old Male With Factor XIII Deficiency: A Case Report

**DOI:** 10.1155/crh/4790198

**Published:** 2025-12-11

**Authors:** Farah Sadiq, Shayan Nawaz, Ali Gohar, Fatima Saleemi, Abdul Rehman Shahid Khan, Masab Ali, Muhammad Husnain Ahmad, Hanzala Zahid, Iqra Nasir

**Affiliations:** ^1^ Department of Internal Medicine, Lahore General Hospital, Lahore, Punjab, Pakistan; ^2^ Department of Internal Medicine, University of Lahore, Lahore, Punjab, Pakistan, uol.edu.pk; ^3^ Department of Internal Medicine, Punjab Medical College, Faisalabad, Punjab, Pakistan, pmc.edu.pk; ^4^ Department of Internal Medicine, S. Tentishev Asian Medical Institute, Kant, Chuy Province, Kyrgyzstan

**Keywords:** Factor XIII deficiency, fresh frozen plasma, intracerebral hemorrhage, rare bleeding diathesis, spontaneous bleeding

## Abstract

Spontaneous, nontraumatic intracerebral hemorrhage (ICH) in young adults with bleeding diathesis, particularly Factor XIII (FXIII) deficiency, is a rare yet life‐threatening condition that affects approximately one‐third of patients. FXIII deficiency typically presents at birth with prolonged umbilical bleeding and can manifest later with recurrent episodes of prolonged bleeding, epistaxis, and bleeding from minor injuries. Diagnosing FXIII deficiency is challenging due to a normal coagulation profile, requiring a detailed clinical history and specialized diagnostic tests for FXIII levels. We report a case of a young male with recurrent spontaneous ICH, frequent epistaxis, and prolonged bleeding from minor trauma. The patient was managed conservatively with fresh frozen plasma (FFP) transfusions and supportive care. Prophylactic FFP infusions were initiated every 2 months to prevent further bleeding episodes, accompanied by regular follow‐up. The patient made a full recovery and is now leading a healthy life without excessive bleeding, demonstrating the importance of early diagnosis and ongoing treatment. This case highlights the need for clinicians to consider FXIII deficiency in patients with unexplained bleeding, particularly when routine coagulation tests are normal, and underscores the value of timely intervention to prevent severe complications like ICH.

## 1. Introduction

Factor XIII (FXIII), also known as fibrin‐stabilizingfactor, plays a critical role in clot stabilization and hemostasis [1, 2]. Deficiency of FXIII is a rare bleeding diathesis that can present in both congenital and acquired forms, leading to impaired clot stability and abnormal bleeding tendencies [1]. Congenital FXIII deficiency is inherited in an autosomal recessive pattern [3], while acquired deficiency can result from immune‐mediated inhibition, heightened consumption, or decreased synthesis of FXIII. Acquired cases may be idiopathic or associated with underlying conditions, such as malignancies (leukemia, adenocarcinoma, and lymphoma) or autoimmune diseases (systemic lupus erythematosus [SLE], rheumatoid arthritis [RA], Henoch‐Schonlein vasculitis [HSV]) [4]. Patients with FXIII deficiency often present with a lifelong bleeding diathesis, which may include symptoms such as easy bruising, subcutaneous bleeding (57%), delayed umbilical cord bleeding (56%), muscle hematomas (49%), menorrhagia, spontaneous miscarriages, and hemorrhage following surgery or trauma (40%) [5, 6]. Central nervous system hemorrhage, including intracranial hemorrhage (ICH), is particularly significant, occurring in approximately 34% of cases and recurring in 30% of these patients. In severe FXIII deficiency, ICH may be the initial manifestation, underscoring the critical nature of early diagnosis [7]. Apart from its role in clot stabilization and hemostasis, FXIII contributes to immunity (by interacting with immune cells and the complement system) and adipogenesis. However, its importance in bone biology, especially in osteoblast differentiation, is currently under study [8].

Diagnosing FXIII deficiency can be challenging because routine coagulation tests such as prothrombin time (PT), activated partial thromboplastin time (aPTT), and platelet function tests typically yield normal results [5]. Definitive diagnosis requires specialized tests, including FXIII activity assays and antigen assays. Quantitative tests include ammonia release assay, amine incorporation assay, and isopeptidase assay, whereas the qualitative method involves clot solubility test (CST) [5]. Imaging studies such as computed tomography (CT) scans and magnetic resonance imaging (MRI) are essential in evaluating patients with recurrent ICH and identifying potential bleeding etiologies.

## 2. Case Description

**Figure 1:**
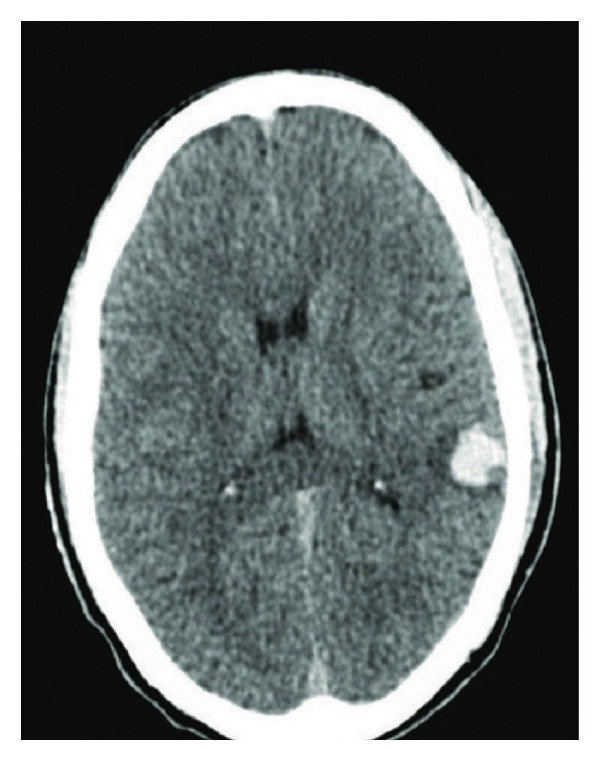
Noncontrast CT scan of the brain showing acute intracerebral hemorrhage in the left temporoparietal region.

A 30‐year‐old single male presented to the medical emergency with a complaint of severe headache associated with multiple episodes of vomiting for two days. There was no history of fever, seizures, any fall, trauma, or drug addiction. There was no past medical history of hepatic, renal, or connective tissue disorder. On physical examination, the patient was hemodynamically stable with Glasgow Coma Scale of 15/15. Pupils were bilaterally reactive to light, and plantars were bilaterally extensors with the following vitals: blood pressure 136/90 mmHg, pulse rate 104 beats per minute, and respiratory rate 17/min. Power was 5/5 in all muscle groups. The rest of the neurological and systemic examination was unremarkable. The past history of the patient revealed that he was diagnosed with FXIII deficiency via quantitative FXIII assay (ammonia release assay) at the age of 12 following evaluation for recurrent spontaneous epistaxis and prolonged bleeding episodes, especially on knees and elbows, after minor traumatic injuries during falls or while playing. Those episodes were not that severe in nature and was settled with first aid during that time. At the time of diagnosis, genetic testing could not be performed due to the unavailability of testing facilities. He had been receiving prophylactic fresh frozen plasma (FFP) infusions every 6 months to reduce the risk of spontaneous bleeding episodes. At the age of 15, he experienced his first spontaneous intraventricular hemorrhage, confirmed by a CT brain scan at that time. During that episode, the patient was managed conservatively with intravenous fluids, mannitol, analgesics, and FFP transfusions. He showed gradual clinical improvement without the need for neurosurgical intervention. He recovered without residual neurological deficits. Consanguinity is noted in the patient’s family history. There was no history of any similar illness in the family. An urgent noncontrast CT scan of the brain revealed an acute intracerebral hemorrhage in the left temporoparietal region (Figure 1). The CT angiogram was normal. His laboratory investigations are summarized in Table 1. Ultrasonography (USG) of the abdomen and pelvis, chest X‐ray, and electrocardiogram were normal. Echocardiography was unremarkable. The patient was admitted on the line of recurrent intracerebral hemorrhage secondary to FXIII deficiency and was managed conservatively with intravenous fluids, FFP transfusions, mannitol, analgesics, and antiemetic. Recombinant FXIII concentrate was not available at our center; therefore, the patient was managed conservatively with FFP transfusions, along with supportive care. As per the neurosurgery team, no neurosurgical intervention was required and the patient was discharged after 5 days of hospital stay.

**Table 1:** Hematological profile and coagulation factor levels.

Test	Result	Reference range
Hemoglobin	13.4 g/dL	13.0–17.0 g/dL
White blood cell count	7800/μL	4000–11,000/μL
Platelet count	307 × 10^9^/L	150–400 × 10^9^/L
Prothrombin time (PT)	12.4 s	11–14 s
Activated partial thromboplastin time (aPTT)	31 s	25–35 s
International normalized ratio (INR)	1.02	0.9–1.1
Factor XIII (quantitative, plasma)	22%	70%–140%
Factor IX	84%	50%–150%
Factor XI	81%	50%–200%

Due to financial constraints, the periodicity of laboratory tests was not maintained. However, he was counseled regarding regular follow‐up in the outpatient department and continuing prophylactic FFP infusions at a dose of 15 mL/kg every 2 months, based on clinical response and availability.

## 3. Discussion

Diagnosing ICH secondary to FXIII deficiency is particularly challenging because standard coagulation tests, such as PT, aPTT, and platelet count, often return normal results [7, 8]. Provisional diagnosis relies heavily on a detailed bleeding history, which may include neonatal umbilical bleeding (a nearly pathognomonic feature), recurrent ecchymoses, postoperative bleeding, spontaneous miscarriages, and delayed wound healing [8]. In contrast to typical presentations, our case was unusual because the patient had no significant bleeding manifestations other than unprovoked childhood epistaxis before experiencing his first episode of intraventricular hemorrhage at the age of 15. This deviation highlights the diagnostic challenges of FXIII deficiency and the necessity of comprehensive investigations for an accurate diagnosis in younger population, as also depicted by Sawlani et al. [9]. It is imperative for neurologists and neurosurgeons to consider FXIII deficiency in patients with unexplained bleeding tendencies despite normal routine coagulation profiles.

In cases of ICH, the intraparenchymal region is the most common site of bleeding, as demonstrated in our patient’s left temporoparietal hemorrhage. This similarity in anatomical presentation to ICH caused by other etiologies suggests that FXIII‐related ICH can mimic common stroke patterns [10, 11]. Interestingly, our patient’s first ICH occurred at 15 years of age, diverging from the typical presentation of congenital FXIII deficiency, where more than 80% of ICHs occur within the first 6 years of life [10].

The patient was managed conservatively with FFP. While FFP and cryoprecipitate remain a viable treatment option, they are not preferred for prophylaxis because they provide variable and relatively low amounts of FXIII, making it difficult to maintain stable levels. Additionally, cryoprecipitate carries pathogen transmission risks and has been withdrawn in many countries [12]. Plasma‐derived FXIII concentrates are now preferred due to advantages such as reduced volume requirements, fewer contaminants, and viral inactivation [7]. Given the high risk of ICH recurrence and the associated morbidity, mortality, and severe neurological complications (e.g., hemiplegia or developmental delays), prophylactic FXIII replacement therapy is strongly recommended. With a half‐life of 5–12 days [8], FXIII prophylaxis is both practical and effective, particularly in patients with a history of recurrent ICH. Especially in pregnant women, where such deficiency leads to recurrent abortions and intrapartum hemorrhages, prophylactic treatment with FXIII concentrate is of extreme importance to alleviate such life‐threatening complications [13].

In our case, FXIII concentrate could not be administered due to nonavailability; hence, FFP was used for both acute management and prophylaxis. Patients with FXIII deficiency and a history of ICH require close monitoring with regular imaging if symptomatic and periodic measurement of FXIII levels to guide prophylactic therapy. Recent evidence supports prophylactic FXIII replacement to maintain trough levels > 5% to prevent bleeding episodes. Plasma‐derived or recombinant FXIII concentrate is typically given at a dose of 35 IU/kg every 4 weeks, which has shown effectiveness in preventing ICH and other major bleeds [5].

FXIII deficiency is inherited in an autosomal recessive pattern, and genetic counseling is important for affected families. Homozygous individuals typically manifest clinically, while heterozygous carriers are usually asymptomatic. Offspring of two carrier parents have a 25% chance of being affected. Early diagnosis in siblings and prenatal counseling may help reduce morbidity associated with delayed recognition [14].

To effectively manage FXIII deficiency in patients with recurrent intracerebral hemorrhage, close laboratory monitoring is essential to ensure adequate and sustained replacement levels. For initial assessment, measure FXIII activity shortly after plasma infusion (approximately 30 min to 1 h) to evaluate the immediate postinfusion or peak level. For trough measurement, assess FXIII levels immediately before the next scheduled infusion to determine the lowest concentration and ensure sufficient maintenance between doses. Following a major bleeding event, such as an intracerebral hemorrhage, more frequent testing (daily or every other day) is recommended to confirm that therapeutic levels are maintained and to guide dose adjustments. Once stability has been achieved, periodic monitoring every 1–3 months is advisable to maintain safe and effective FXIII levels. Because plasma infusions typically produce smaller increases in FXIII levels compared to cryoprecipitate or purified concentrates, more frequent infusions or higher doses may be required. Transitioning to a more concentrated product should be considered if plasma alone fails to maintain target levels. Adjustment of infusion dosage or frequency is based on serial FXIII measurements and the patient’s clinical response to ensure optimal replacement. If plasma therapy proves inadequate, switching to cryoprecipitate or specific FXIII concentrates may provide more reliable and sustained correction. Bleeding Prevention: Consistent laboratory monitoring is crucial to maintaining FXIII levels within the therapeutic range, thereby minimizing the risk of spontaneous or recurrent hemorrhage [15–17].

## 4. Conclusion

Our case highlights the importance of screening and testing of clotting factors in young patients with recurrent bleeding problems and a history of consanguinity to prevent complications in cases with late presentation. Early detection of disease and plasma treatment can help in preventing morbidity and mortality. The significance of this case lies in emphasizing the need for advanced research and the establishment of specialized diagnostic facilities for clotting factor deficiencies and other rare bleeding diathesis. It also highlights the importance of increasing physician awareness of clotting factor deficiencies as a differential diagnosis in patients with extensive bleeding and delayed hemostasis.

## Ethics Statement

Ethical approval was not required for this case report.

## Consent

A written informed consent was obtained from the patient based on the journal’s policies.

## Conflicts of Interest

The authors declare no conflicts of interest.

## Author Contributions

Farah Sadiq: conceptualization; data curation; project administration; supervision; validation; visualization; writing–original draft; and writing–review and editing.

Shayan Nawaz: project administration; supervision; validation; visualization; writing–original draft; and writing–review and editing.

Ali Gohar: project administration; supervision; validation; visualization; writing–original draft; and writing–review and editing.

Fatima Saleemi: validation; visualization; writing–original draft; and writing–review and editing.

Abdul Rehman Shahid Khan: validation; visualization; writing–original draft; and writing–review and editing.

Hanzala Zahid: validation; visualization; and writing–review and editing.

Masab Ali: validation; visualization; writing–original draft; and writing–review and editing.

Muhammad Husnain Ahmad: validation and visualization.

Iqra Nasir: writing–review and editing.

## Funding

The authors did not receive any funding for this work.

## Data Availability

Data and materials are available upon request from corresponding author.
